# Nonlinear dispersion relation in integrable turbulence

**DOI:** 10.1038/s41598-022-14209-7

**Published:** 2022-06-20

**Authors:** Alexey Tikan, Félicien Bonnefoy, Guillaume Ducrozet, Gaurav Prabhudesai, Guillaume Michel, Annette Cazaubiel, Éric Falcon, Francois Copie, Stéphane Randoux, Pierre Suret

**Affiliations:** 1 Univ. Lille, CNRS, UMR 8523, PhLAM - Physique des Lasers Atomes et Molécules, F-59000 Lille, France; 2 École Centrale de Nantes, LHEEA, UMR 6598, CNRS, 44 321 Nantes, France; 3 LPENS, Ecole Normale Supérieure, CNRS, PSL Research University, Sorbonne Université, Université Paris Cité, F-75005 Paris, France; 4 Institut Jean Le Rond d’Alembert, UMR 7190, CNRS, Sorbonne Université, 75 005 Paris, France; 5 Université Paris Cité, CNRS, MSC, UMR 7057, F-75013 Paris, France; 6 Present Address: Institute of Physics, Swiss Federal Institute of Technology Lausanne (EPFL), 1015 Lausanne, Switzerland

**Keywords:** Physics, Fluid dynamics, Statistical physics, thermodynamics and nonlinear dynamics

## Abstract

We investigate numerically and experimentally the concept of nonlinear dispersion relation (NDR) in the context of partially coherent waves propagating in a one-dimensional water tank. The nonlinear random waves have a narrow-bandwidth Fourier spectrum and are described at leading order by the one-dimensional nonlinear Schrödinger equation. The problem is considered in the framework of integrable turbulence in which solitons play a key role. By using a limited number of wave gauges, we accurately measure the NDR of the slowly varying envelope of the deep-water waves. This enables the precise characterization of the frequency shift and the broadening of the NDR while also revealing the presence of solitons. Moreover, our analysis shows that the shape and the broadening of the NDR provides signatures of the deviation from integrable turbulence that is induced by high order effects in experiments. We also compare our experimental observations with numerical simulations of Dysthe and of Euler equations.

## Introduction

*Nonlinear dispersion relation* (NDR) ( i.e. the relation between wavevectors *k* and angular frequencies $\omega$ for waves of finite amplitude) is a powerful tool to highlight dynamical and spectral properties of nonlinear wave fields^1–3^. The concept of NDR is relevant to all the fields of Physics where the Fourier modes provide a relevant basis to describe the waves and their interactions (nonlinear optics^4^, hydrodynamics^2,3,5^, hydroelasticity^6^, mechanics^1^, quantum optics^7^, plasma physics^8,9^,...). In particular, it has been extensively used in the context of wave turbulence (WT), where the interaction between random nonlinear waves is dominated by resonances among random Fourier components^1–3,10–12^.

On the other hand, systems of nonlinear random waves governed by integrable partial differential equations such as the Korteweg de Vries, sine-Gordon or the one-dimensional nonlinear Schrödinger equation (1DNLSE) represent a profoundly different class of problems because the natural basis for the analysis is provided by the inverse scattering transform (IST) sometimes called “nonlinear Fourier transform”^13^. In this framework, the field is decomposed into two components—the radiation and the solitons- identified with two types of nonlinear spectra—the continuous spectrum and the discrete spectrum respectively^14^.

Integrable wave systems exhibit a remarkable form of turbulence called “integrable turbulence” (IT)^15–19^. In particular, while exact and non trivial resonances play a crucial role in WT, they are not allowed in IT (see^20^ and “Methods”). Moreover, in WT, spectra are often characterized by power laws while the known spectra in IT exhibit exponential tails^21^. Soliton gas recently investigated in water waves experiments represents a peculiar “purely solitonic” case of IT^22–24^. However, IT more generally involves the interplay between nonlinear radiation (dispersive waves) and solitons. While these two components are naturally distinguished within the framework of IST, they cannot be easily separated in the physical space. Even if IT and WT regimes are of strongly different natures, the possibility to analyse IT and to reveal the existence of solitons by using conventional tools such as the NDR (instead of the complicated machinery of IST) is of crucial importance from the practical and fundamental point of views^23^.

Surprisingly, despite the universality of the 1DNLSE, little attention has been paid to its NDR up to a very recent theoretical study^25^ in which various kinds of initial conditions have been investigated. In this interesting work, the authors show that the NDR of weakly nonlinear random waves only experiences the well-known frequency shift^26,27^, while the NDR of a single soliton is a straight line having a slope corresponding to its group velocity (see “Methods”)^25,28^. To the best of our knowledge, the concept of NDR has not been applied to IT experiments described at leading order by 1DNLSE. In particular the (Fourier) spectral signature of solitons has not been reported in this context.

In this manuscript, we first use numerical simulations of 1DNLSE to investigate the NDR and the power spectral density -PSD- (in the space-time $(\omega ,k)$ Fourier plane) in IT. We show that the PSD and the NDR present clear signature of the growing influence of solitons when the nonlinearity strength is increased. We then report on an experiment in which partially coherent (random) deep water surface gravity waves propagate along a long one-dimensional flume. We demonstrate that removing the carrier wave enables the accurate measurement of the NDR of wavefields having a narrow Fourier spectrum by using a very limited number of gauges. In experiments, the NDR is found to reveal both the existence of solitons embedded in the random field and a deviation from IT induced by higher-order nonlinear effects not taken into account in the 1DNLSE model. Simulations of Euler and Dysthe equations confirm the influence of high order terms and provide a deeper insight into the mechanisms behind the formation of coherent structures observed in the experiment.

## Results

### Numerical simulations (nonlinear Schrödinger equation)

Considering unidirectional deep water gravity waves having a narrow spectrum, the surface elevation $\eta (\tau ,z)$ is:

1 $$\begin{aligned} \eta (\tau ,z)=\frac{1}{2}\left( \psi _{r}(\tau ,z)e^{i (k_0z -\omega _0 \tau )}+c.c.\right) ; \end{aligned}$$

where $\psi _{r}$ is the slowly-varying complex envelope, $f_0=\omega _0/(2\pi )$ and $k_0=\omega _0^2/g$ are the frequency and the modulus of the wavevector of the carrier wave respectively, *z* is the propagation distance and $\tau$ is the time measured in the laboratory frame. For deep water gravity waves, the dynamics of $\psi (t,z)=\psi _{r}(\tau ,z)$ is described at leading order by the focusing 1DNLSE^29^:

2 $$\begin{aligned} i\frac{\partial \psi }{\partial z}= \frac{1}{g}\frac{\partial ^2 \psi }{\partial t^2}+\gamma |\psi |^2\psi , \end{aligned}$$

where $t=\tau -z/c_g$, $c_g=g/(2 \omega _0)$ is the group velocity evaluated at the frequency $f_0$, $\gamma =k_0^3$ and *g* is the gravity acceleration. In simulations and in experiments, the initial conditions are partially coherent waves produced from the linear superposition of numerous independent Fourier components having a Gaussian spectrum (see “Methods” and^19,21,30^). It is useful to introduce the degree of nonlinearity of the wave propagation, which is given by the parameter $\Gamma$:

3 $$\begin{aligned} \Gamma =\frac{\gamma g P_0}{(2 \pi \Delta f)^2}\;\;\text {with } P_0=\langle |\psi (t,z=0)|^2 \rangle \end{aligned}$$

where $\Delta f\ll f_0$ is the initial spectral bandwidth evaluated at $z=0$ and where $\langle ... \rangle$ denotes the averaging over time and/or realizations. Note that $\langle |\psi |^2\rangle =2 \langle \eta ^2\rangle$ and that $\Gamma =BFI^2$ where BFI is the Benjamin-Feir Index^31,32^ (see “Methods”). It has been shown that large values of $\Gamma$ lead to the formation of rogue waves characterized by heavy-tailed statistical distributions of the surface elevation^16,19,30,31,33–35^.

**Figure 1:**
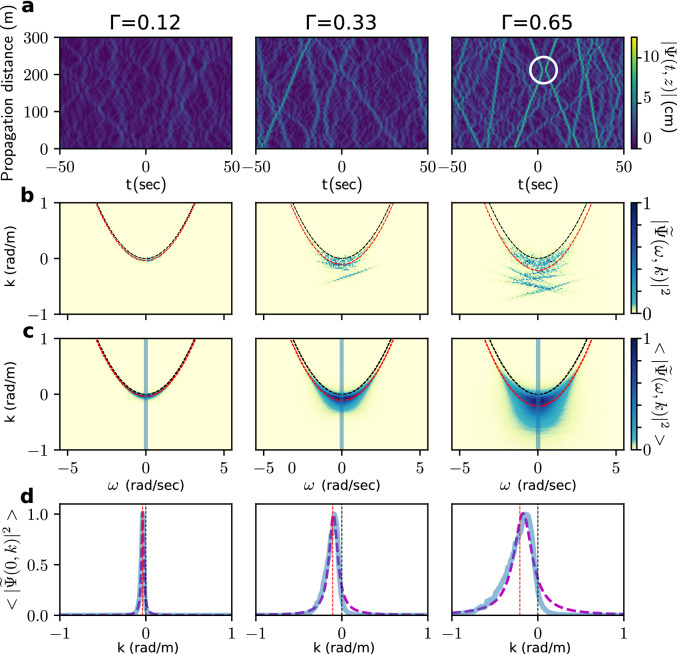
Numerical simulations of the 1-D NLS equation. Three columns correspond to three different values of $\Gamma$ 0.12, 0.33, and 0.65, respectively. The central frequency of the carrier wave and the initial width of the wave spectrum are set to $f_0=1.15$ Hz and $\Delta f = 0.2$ Hz. **(a)** Spatiotemporal diagram for the complex envelope amplitude $|\psi (t,z)|$. **(b)** Corresponding nonlinear dispersion relation $|{\widetilde{\psi }}(\omega ,k)|^2$ normalized to the maximum. **(c)** Nonlinear dispersion relation averaged over 1000 realizations and normalized to the maximum. Propagation distance is 500 m. **(d)** Cross-section of the averaged nonlinear dispersion relation at $\omega =0$ (along the blue line in **c**). Dashed purple line shows a Lorentzian fit. In **(b–d)**, vertical black and red dashed lines represent the linear dispersion $k(\omega )$ and its nonlinear correction of Eq. (5) respectively.

We have first performed numerical simulations of Eq. (2) for three values of $\Gamma$ (see “Methods” for details). Fig. 1a, represents the typical spatio-temporal dynamics of IT developing from partially coherent waves in the focusing regime of 1DNLSE. Remarkably, the number of isolated pulses emerging in the random wave field increases with the nonlinearity. Note that our numerical simulations reveal the presence of elastic collisions (see for the example the white circle in Fig. 1), a signature of solitons in integrable systems^13^.

We define the space-time double Fourier transform as:

4 $$\begin{aligned} {\widetilde{\psi }}(\omega ,k)=\iint \psi (t,z)\,e^{+i\omega t}\,e^{-i k z}\,dt\,dz \end{aligned}$$

The power spectrum (PSD) $|{\widetilde{\psi }}(\omega ,k)|^2$ of *one* realization of $\psi (z,t)$ is plotted in blue in Fig. 1b. As pointed out in^25^ and reported below in “Methods”, the straight lines in the $k-\omega$ space are signature of the solitons observed in the spatio-temporal dynamics (Fig. 1a). Note that the theoretical description of the statistical distribution of the slopes of the straight lines—associated to the velocities of the solitons—is a theoretical open question. We also plot the spectrum $\langle |{\widetilde{\psi }}(\omega ,k)|^2\rangle$ averaged over several realizations in Fig. 1c. The linear dispersion of 1DNLSE reads $k(\omega )=\omega ^2/g$^36^ and is plotted in dashed black lines in Fig. 1b,c. The NDR can be defined as the peak wavevectors ${\tilde{k}}$ of the PSD for each value of $\omega$ in the $(\omega ,k)$ plane^25^. For moderate nonlinearities and narrow Fourier spectra, the nonlinear NDR gets shifted from the linear one and reads^25,36^:

5 $$\begin{aligned} {\tilde{k}}(\omega ,P_0)=k(\omega )-2 \gamma P_0. \end{aligned}$$

As expected from Eq. (5), the NDR computed from numerical simulations shifts toward negative values of *k* (see red dashed lines in Fig. 1b,c). The Fig. 1c,d show that, the PSD broadens around the NDR when $\Gamma$ increases because of the energy exchange among Fourier modes. This broadening phenomenon is well-known in standard WT with resonant interactions^1–3,37^ but, to the best of our knowledge, it has not been reported in IT where resonances are forbidden (see “Methods”).

The $k-$spectrum at $\omega =0$, i.e. $n_k=\langle |{\widetilde{\psi }}(0,k)|^2\rangle$ is plotted in Fig. 1d. For small values of $\Gamma$, the maximum of this curve coincides with the value predicted by the weakly nonlinear theory (i.e. Eq. (5), red dashed line in Fig 1d). Note that, in this weakly nonlinear regime, $n_k$ can be empirically fitted by a Lorentzian distribution (purple dashed line in Fig. 1d). To the best of our knowledge, this remarkable fact, not known yet in IT, has not been described theoretically. At higher nonlinearities, the number of straight lines associated with solitons (individually observed in Fig. 1a,b for $\Gamma =0.65$) increases. The NDR of a single soliton follows the straight line $k=c_s\,\omega +(k_s-c_s\omega _s-\frac{1}{2}\gamma |\psi _0|^2)$ where $c_s$ is the group velocity of the soliton in the (*t*, *z*) plane, $\omega _s$ is the central frequency of the soliton and $k_s=k(\omega _s)=\omega _s^2/g$ follows the linear dispersion (see^25^ and “Methods”). As a consequence, the nonlinear phase shift acquired by solitons during their propagation places the solitonic lines well below the dispersion parabola described by Eq. (5) (see Fig. 1b).

Consequently, the strong and asymmetric broadening of the PSD profile $n_k$ toward negative values of *k* (Fig. 1d) can be considered as a signature of the increasing number of solitons in the strongly nonlinear regime of IT.

### Experiments

**Figure 2:**
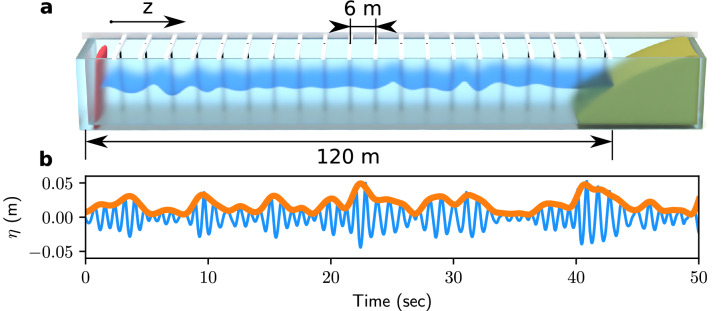
Experimental facility. **(a)** Schematic representation of the 120m-long water tank facility at École Centrale de Nantes. The surface elevation is recorded by a set of probes placed every 6 m of the water tank length. **(b)** Typical experimental wave train (surface elevation $\eta$, blue line) and its envelope (orange line) reconstructed by using the Hilbert transform.

In order to investigate experimentally the NDR described above, we have used the setup described in^38^ and schematically shown in Fig. 2a. Regimes close to IT can be achieved in unidirectional deep water waves having a narrow Fourier spectrum. Such waves are generated at one end of a 148 m long, 5 m wide and 3 m deep wave flume by a computer-assisted flap-type wavemaker (see Fig. 2a). The flume is equipped with an absorbing device strongly reducing wave reflection at the opposite end^38^. The surface elevation $\eta (\tau ,z)$ is measured by using 20 equally spaced resistive wave gauges that are installed along the water tank at distances $z_j=6\,j$ m, $j=1,2,...20$ from the wavemaker located at $z=0$ m. This provides an effective measuring range of 120 m and a resolution of the $k-$spectrum of $2\pi /120$ rad m$^{-1}$ (see “Methods”). The envelope $\psi (t,z=0)$ of the surface elevation has a central frequency $f_0=1.15$ Hz and it is designed using the same procedure as the one used in our numerical simulations, i.e. by performing the linear superposition of a large number of independent random Fourier modes. The degree of nonlinearity is varied by changing either the averaged amplitudes or the initial spectral width $\Delta f$ of the waves generated in the water tank.

**Figure 3:**
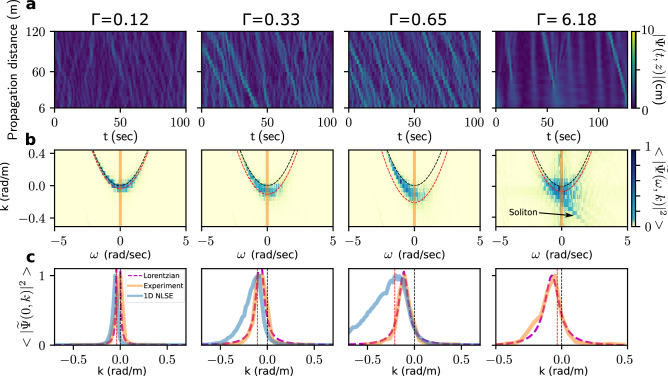
Experimental reconstruction of the nonlinear dispersion relation. Four columns correspond to four different values of $\Gamma$ 0.12, 0.33, 0.65, and 6.18, respectively. **(a)** Spatiotemporal diagram of the wave envelope amplitude $|\Psi (t,z)|$. Data received from 20 probes have been post-processed and arranged in 20 vertical rows subtracting waves’ group velocity ($t=\tau -z/c_g$). **(b)** Nonlinear dispersion relation reconstructed from the evolution of the complex wave envelope. $\Gamma =$ 0.12, 0.33, 0.65 correspond to an initial spectral width $\Delta f=0.2$ Hz and $\langle |{\widetilde{\psi }}(\omega ,k)|^2\rangle$ is averaged over several realizations (see “Methods”). In order to observe the signature of a single soliton, the NDR is not averaged for $\Gamma =6.18$ (corresponding to $\Delta f=0.037$ Hz). **(c)** Cross-section of the nonlinear dispersion relation at $\omega =0$. Dashed purple line shows a Lorentzian fit, blue line shows corresponding results of NLS simulation.

A typical temporal evolution of the surface elevation experimentally recorded at the first gauge ($z=6$ m) is plotted in the Fig. 2b. The slowly varying amplitude $\psi (z,t)$ is determined by using Hilbert Transform following techniques described, e.g., in Ref.^29^. Typical spatio-temporal evolution of $|\psi |$ is plotted in Fig. 3a where we use the retarded time $t=\tau -z/c_g$. When $\Gamma$ increases, we observe the emergence of pulses localized in space and time. The emerging pulses become narrower and most of them achieve a negative speed in the (*t*, *z*) diagram (see below).

As expected from our numerical simulations, the experimental PSD broadens and shifts toward the negative values of *k* (Fig. 3b). However, at high nonlinearity, the measured NDR deviates significantly from the numerical simulations and becomes asymmetric with $\omega$. This phenomenon is the well-known “frequency downshift” of surface gravity waves induced by high order nonlinearities responsible for the negatives speeds observed in the (*t*, *z*) diagram shown in Fig. 3a^39^. The numerical simulations of Dysthe equation (see below) and of the Euler equations (see Supplementary Material) confirm that this shape of the NDR is indeed induced by effects (not included in 1DNLSE) which break integrability of the wave equation.

### Simulations of the Dysthe equation

The 1DNLSE (Eq. 2) is established under the assumptions of weak nonlinearity of the wave field as well as the narrowbandedness of its energy content. As it could be expected, at high values of the nonlinear parameter $\Gamma$, the measured NDR deviates from the one computed by performing numerical simulations of the 1DNLSE. The Dysthe equation (a higher-order nonlinear and not integrable generalized version of the 1DNLSE) provides a simple model that reproduces qualitatively the $\omega$ asymmetry of the NDR observed in experiments. Dysthe equation can be expressed as follows^40^:

6 $$\begin{aligned} \frac{\partial \psi }{\partial z} + \frac{i}{g}\frac{\partial ^2 \psi }{\partial t^2} + i k_0^3 |\psi |^2\psi = \frac{k_0^3}{\omega _0} \left[ \underbrace{6|\psi |^2\frac{\partial \psi }{\partial t}}_{\rm {a}} + \underbrace{2\psi \frac{\partial |\psi |^2}{\partial t}}_{\rm {b}} - \underbrace{i\psi {\mathscr {H}}\left( \frac{\partial |\psi |^2}{\partial t}\right) }_{\rm {c}} \right] , \end{aligned}$$

where ${\mathscr {H}}$ stands for the Hilbert transform defined as follows:${\mathscr {F}}({\mathscr {H}}(f(t))) = -i\,\mathrm {sign}(\omega ){\mathscr {F}}(f(t))$, where ${\mathscr {F}}$ represents the Fourier transform and $\mathrm {sign}$ is the signum function. The extra terms labelled ’a’, ’b’ and ’c’ in Eq. (6) represent higher order terms in a perturbative approach of Euler equation where the small parameter is the spectral width $\Delta f$. If $\Delta f\rightarrow 0$, the derivatives terms ’a’, ’b’ and ’c’ vanish whereas the dominant cubic term $i k_0^3 |\psi |^2 \psi$ remains unchanged.

**Figure 4:**
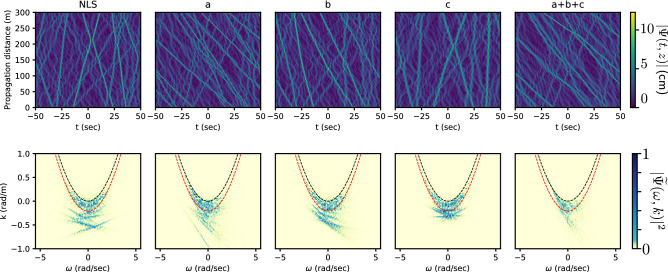
Numerical simulation of Dysthe model. Parameters correspond to Fig. 1a of the manuscript $\Gamma$ = 0.65, $\varepsilon$ = 0.14 (see “Methods”). Label ’NLS’ corresponds to simulations of 1DNLSE. Labels ’a’, ’b’, ’c’, and ’a $+$ b $+$ c’ indicate terms of Eq. (6) are added to the 1DNLSE core.

In order to investigate the influence of additional terms present in Dysthe equation, we have simulated the nonlinear propagation of identical initial conditions used in Fig. 1a, $\Gamma$ = 0.65, including Dysthe terms labeled ’a’, ’b’, and ’c’ separately as shown in Fig. 4. Parameters used in the numerical simulations are the same as those used for the numerical integration of the 1DNLE reported above.

As one can see in Fig. 4, the derivatives included in the terms ’a’ and ’b’ contribute to the nonlinear spectral shift, leading to the change of solitons’ group velocity (asymmetry in $\omega$). The term ’c’ reduces the shift of solitons leading to a picture similar to 1DNLSE but with a smaller amplitudes of the localized structures which can be seen in the corresponding NDR plots.

Importantly, we have also run numerical simulations Euler equations by using high-order spectral (HOS) method (see Supplementary Material). The results of these simulations are close to those obtained with the Dysthe equation.

### Nonlinear shift and broadening of the nonlinear dispersion relation

Our data analysis reveals that higher-order effects discussed above reduce the number of solitons embedded in random waves: indeed, we found that for $\Gamma \le 1$, the straight lines in the $(\omega ,k)$ space—signatures of solitons—appear much less frequently than in 1DNLSE simulations. Nevertheless, we have also observed these spectral signatures of solitons at extremely high nonlinearity (which is achieved with small values of the $\Delta f$, see the fourth column of Fig. 3).

The shapes of the PSD and of the NDR provide another signature of the deviation from IT: while the NDR predicted from the 1DNLSE becomes asymmetric at high nonlinearity, the experimental PSDs profiles $n_k=\langle |{\widetilde{\psi }}(0,k)|^2\rangle$ in Fig. 3c coincide with a Lorentzian fit for all values of $\Gamma$, a result also observed for HOS simulations.

**Figure 5:**
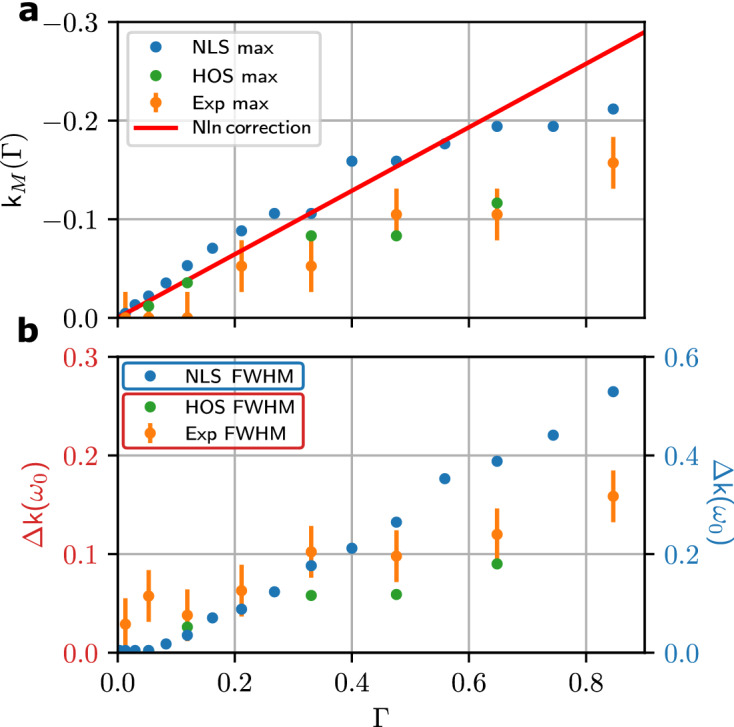
Quantitative comparison of the experimental results with different numerical models as a function of $\Gamma$. **(a)** Value of *k* at the NDR maximum for $\omega =0$. Blue and green dots correspond to NLS and HOS simulations; orange dots show experimental data. Red line represents the theoretical curve (Eq. 5). **(b)** Full width at half maximum of the NDR for $\omega =0$. Note that the vertical scale is different for experiments/HOS simulations (left scale) and 1DNLSE simulations (right scale). For all points in (**a,b)**, f$_0$ = 1.15 Hz, $\Delta$f = 0.2 Hz.

The influence of the nonlinearity and of high order effects can be quantified with the help of the position $k=k_M(\Gamma )$ of the maximum and of the full width at half maximum $\Delta k(\Gamma )$ of $n_k$ (see Fig. 5a,b respectively). Figure 5a shows that, both in experiments and simulations, $k_M(\Gamma )$ evolves more slowly than predicted by the weakly nonlinear theory—see Eq. (5). Moreover, because of the smaller number of solitons, the shift of the NDR toward negative values of *k* is weaker in experiments and HOS simulations than in IT (1DNLSE simulations).

## Discussion

Our work provides new insight into the spectral properties of unidirectional nonlinear random waves. Our numerical simulations of the 1DNLSE show how the number of emerging solitons is directly related to the strength of nonlinearity in IT. Moreover, while a previous study has focused on the position of the maxima of the NDR^25^, we also investigate the broadening of the NDR. Simulations reveal that the NDR provides a spectral signature of the solitons embedded in the random waves (asymmetric broadening in the *k* direction at high nonlinearity). Further investigations are needed to establish the theoretical link between the nonlinear spectra computed in the framework of IST and the broadening of the NDR (Fourier spectra).

From the experimental point of view, by focusing our analysis on the slowly-varying amplitude $\psi$, we were able to measure very accurately slight deviations from the predicted nonlinear dispersion relation. The results reported here provide new insights into an old fundamental problem of hydrodynamics: the measurement of the dispersion relation of random surface gravity waves (see for example^5^ and refs. therein). Various theoretical and experimental works have been devoted to this question, see e.g.^5,36,41–47^. For water tanks equipped with transparent side walls, cameras may be used to record the full spatio-temporal dynamics^23^. Otherwise, a large number of gauges is needed^5^. The central point of our strategy is to remove the carrier wave frequency in order to retrieve the NDR of the slowly varying amplitude of waves having a narrow spectrum. Our simple experimental technique can be easily implemented in further investigations of the NDR of unidirectional water waves by using a very limited number of probes (20 probes here instead of 384 probes in^5^ for example, see “Methods”).

Our work contributes to the understanding of the NDR in nonlinear waves systems that involve solitons. Recently, a non trivial NDR has been established for finite gap solutions in the context of NLS soliton gases^48^. The possible relationship between this NDR derived for soliton gases and the Fourier NDR studied here is an open fundamental question. On another side, the concept of NDR is receiving interest in the photonics community, where it has been for example recently used to explain the effect of dissipative soliton hopping in a photonic dimer^49^ or to describe noise properties of a soliton frequency comb in a synchronously pumped cavity^50^. In this article, we have demonstrated that the NDR measured in deep water waves experiments is close to the one predicted by using the 1DNLSE for low nonlinearity. For high nonlinearity, the measured NDR exhibits a signature of solitons but experiments deviates from IT because perturbative effects not taken into account in the 1DNLSE limit the emergence of solitons. It has been demonstrated that optical fiber experiments can be very close to integrability for high nonlinearity^19,30^. The measurement of the NDR in optical fibers is extremely challenging but it has been recently demonstrated in a double loop fiber devices^51^. We hope that our work will also stimulate further investigation of the NDR of IT in photonics.

## Methods

### Nonlinear Schrödinger equation (1DNLSE)

#### Strength the nonlinearity: Benjamin-Feir index

In order to compare nonlinearity and group velocity dispersion in the framework of the 1DNLSE (Eq. 2), it is useful to introduce a linear and a nonlinear propagation length as follows:

7 $$\begin{aligned} z_{lin}=\frac{g}{(2\pi \Delta f)^2}\;\;\ \mathrm{and}\;\;\;\; z_{nlin}=\frac{1}{\gamma P_0}, \end{aligned}$$

where $\Delta f$ is a typical initial spectral bandwidth and $P_0=\langle |\psi (t,z=0)|^2\rangle$ where $\langle ... \rangle$ is the averaging over time and/or realizations. The degree of nonlinearity of the wave propagation is given by the parameter $\Gamma =z_{lin}/z_{nlin}$ (see Eq. 3).

Note that in the context of ocean waves, $\Gamma =\text {BFI}^2$ where BFI is the Benjamin-Feir Index^31,32^. BFI index can also be expressed as follows:

8 $$\begin{aligned} \text {BFI} = \frac{\varepsilon }{(\Delta f /f_0)}=\sqrt{\Gamma }, \end{aligned}$$

where $\varepsilon = k_0 \sqrt{2} \sigma$ is the wave steepness with $\sigma ^2=\langle \eta ^2\rangle =\langle |\psi |^2\rangle /2$ and $\Delta f$ and $f_0$ are the average spectral width and the central frequency of the initial wave packets respectively.

### Numerical simulations of 1DNLSE

In simulations and in experiments, the initial conditions are partially coherent waves made of the linear superposition of independant Fourier components at $z=0$ and read:

9 $$\begin{aligned} \psi (t,z=0)=\psi _0 \sum _{l=-N/2}^{+N/2} e^{-\frac{1}{2}(f_l/\Delta f)^2} e^{i \,2 \pi \,f_l\,t} e^{i\phi _l} \end{aligned}$$

where $f_l=l/T_{max}$, $T_{max}$ is the temporal duration of the experiments and $\phi _l$ are independently and randomly distributed over $[0,2\pi ]$. Real part and imaginary part of such partially coherent waves exhibit Gaussian statistics at $z=0$ (see^21^ for details).

Along the propagation in the focusing regime of 1DNLSE, the statistics deviates from Gaussianity and the probability density function of the wave amplitude becomes heavy-tailed. Note that the statistical characteristics of partially coherent waves are very different than plane waves initially perturbed by noise which is also investigated in^25^. For a comparison between the two cases, refer e.g. to^52^.

Numerical simulations of Eq. (2) are realized using step-adaptive high order Runge-Kutta method. We construct different initial conditions using the random phase approach where a uniformly distributed phase is added to every Fourier component of a Gaussian spectrum with $\Delta f=$ 0.2 Hz. Typical temporal windows used in the numerical simulations correspond to 100 seconds. Three parameters of simulations depend on the value of the steepness: the number of points *N*, the length of propagation $L_{max}$ and the number of realizations $N_{sample}$. We separate the numerical studies into three ranges of steepness $\varepsilon$ (see Table 1).

**Table 1:** Parameters of numerical simulations of 1DNLSE.

Ranges of $\varepsilon$	*N*	$L_{max} (m)$	$N_{sample}$
$\varepsilon \le 0.05$	2048	500	10,000
$0.05<\varepsilon \le 0.09$	2048	500	500
$0.09<\varepsilon \le 0.19$	1024	2000	100

In order to reconstruct numerically NDR, we multiply the spatiotemporal diagram by a Super-Gaussian window with power 15 along z direction avoiding thereby undesirable effects related to the Fourier analysis of non-periodic signals.

### Integrable turbulence: absence of non trivial resonances in the 1DNLSE

In general, WT is described by the resonant interactions among the Fourier components of the wave field^53,54^. On the contrary, the non trivial resonances are forbidden in integrable turbulence^20,55^. We consider the third order nonlinear interaction of four monochromatic waves $i=1,2,3,4$ of pulsation $\omega _i$ and wavenumber $k_i(\omega _i)$ in a unidirectional dispersive media. In this context, the resonances conditions of four wave mixing in the standard wave turbulence read $\omega _1+\omega _2=\omega _3+\omega _4$ and $k_1+k_2=k_3+k_4$ where $k_i=k(\omega _i)$ satisfies the *linear* dispersion relation $k(\omega )$. The linear dispersion relation of deep water waves is $k(\omega )=\omega ^2/g$ and exact resonances thus lead to:$\omega _1^2+\omega _2^2=\omega _3^2+(\omega _1+\omega _2-\omega _3)^2 \text { and then to : } \omega _1(\omega _3-\omega _2)=\omega _3(\omega _3-\omega _2)$. Finally, $\omega _1=\omega _3$ and $\omega _2=\omega _4$ or $\omega _2=\omega _3$ and $\omega _1=\omega _4$ *i.e.* exact resonances of non trivial interactions ($\omega _1\ne \omega _3$ and $\omega _1\ne \omega _4$) are forbidden.

### Nonlinear dispersion of an isolated soliton

The fundamental soliton solution of the 1DNLSE (Eq. 2) reads^56^:

10 $$\begin{aligned} \psi _S(t,z)=\psi _0 \, \mathrm{sech} \big [(t-c_s\,z)/\tau \big ] \times \exp \big [i\big (k_s z-\omega _s t\big )\big ]\,\exp \big [-i A z\big ] \end{aligned}$$

where the duration of the soliton is $\tau =\sqrt{2/(\gamma g |\psi _0|^2)}$. $k_s=k(\omega _s)$ obeys the linear dispersion relation, $c_s=c(\omega _s)=dk/d\omega =2\omega _s/g$ is the group velocity of the soliton in the (*z*, *t*) plane and $A=\gamma |\psi _0|^2/2$. The double Fourier transform of $\psi _S(t,z)$ is given by:

11 $$\begin{aligned} \widetilde{\psi _S}(\omega ,k)=2\pi ^2\tau \, \psi _0 \, \mathrm{sech}\big [(\omega -\omega _s) \pi \tau /2\big ]\times \delta \bigg [k-\big (k_s+(\omega -\omega _s) c_s-A\big ) \bigg ] \end{aligned}$$

As a consequence, the NDR of a single soliton follows the straight line $k=c_s\,\omega +(k_s-c_s\omega _s-\frac{1}{2}\gamma |\psi _0|^2)$^25^.

## Measurement of the nonlinear dispersion relation

### Resolution of the measurement of *k*

The key point in our approach is to remove the carrier wave before computing the NDR. This allows us to reveal the details of the NDR of the slowly varying envelop. In the water tank, the gauges are separated by 6 m and the maximum measurable wavevector is around 1.05 m$^{-1}$. As a consequence, contrary to Taklo et al. who use 384 probes, we do not resolve the wavevectors of the carrier wave $2\pi /\lambda _0\simeq 5.3$ m$^{-1}$ and of the harmonics. Our strategy enables the accurate measurement of the NLDR of the slow varying envelop of the wave by using only 20 probes. This provides an effective measuring range of 120 m with a resolution of the measurement of the $k-$spectrum of $\Delta k_{min}=2\pi /120$ rad m$^{-1}$. Note finally that, in^5^, the accuracy of the measurement of *k* given by the length of the water tank is $\Delta k_{min}/k_0=0.014$ while our setup enables an accuracy of $\Delta k_{min}/k_0 < 0.01$.

In order to measure the averaged spectra and NDR, we use 3, 6 and, 3 experimental runs with a duration of 512 s for $\Gamma =0.12$, 0.33, 0.65, respectively. One run of 128 s have been used for $\Gamma =6.18$.

Note that in NLS and HOS (high-order spectral, see Supplementary Material) simulations, the chosen lengths of propagation depend on the parameters and vary typically from 300 to 500 m. The uncertainty of measurement of $k_M$ and $\Delta k$ is therefore significantly lower in simulations than in experiments.

### Evaluation of the full width at half maximum $\Delta k$ of the position of the maximum $k_M$

In Fig. 4, we report the evaluation of the full width at half maximum $\Delta k$ and of the position of the maximum $k_M$ of the function $f(k)=|{\widetilde{\psi }}(k,\omega =0)|^2$ in 1DNLSE, HOS simulations and in experiments. The accuracy of the measurement of $\Delta k$ and $k_M$ is limited both by the discretization of *k* (see above the uncertainty $\Delta k_{min}$) and by the random fluctuations of *f*(*k*). In order to overcome these difficulties, when it is appropriate, we evaluate $\Delta k$ and $k_M$ by using best fitting procedure with Lorentzian function.

## Supplementary Information

Supplementary Information.

## Data Availability

The datasets generated and/or analysed during the current study are available in the *Figure data: Nonlinear dispersion relation in integrable turbulence* repository, https://zenodo.org/record/6595429.
