# Supplementary material for: Nonlinear dispersion relation in integrable turbulence
Source: Sci Rep. 2022 Jun 20;12:10386. doi: 10.1038/s41598-022-14209-7 (PMC9209409; doi:10.1038/s41598-022-14209-7)
Supplement: Supplementary file 1 — Supplementary Information. [file 41598_2022_14209_MOESM1_ESM.pdf]

Nonlinear dispersion relation in integrable turbulence: Supplementary Material

Alexey Tikan[1,2], Félicien Bonnefoy[3], Guillaume Ducrozet[3], Gaurav Prabhudesai[4], Guillaume Michel[5], Annette Cazaubiel[6], Éric Falcon[6], Francois Copie[1], Stéphane Randoux[1], and Pierre Suret[1,*]

[1]Univ. Lille, CNRS, UMR 8523 -PhLAM - Physique des Lasers Atomes et Molécules, F-59000 Lille, France
[2]Present address: Institute of Physics, Swiss Federal Institute of Technology Lausanne (EPFL), CH-1015 Lausanne, Switzerland
[3]École Centrale de Nantes, LHEEA, UMR 6598 CNRS, F-44 321 Nantes, France
[4]LPS, ENS, CNRS, Univ. Pierre et Marie Curie, Univ. Paris Diderot, F-75 0005 Paris, France
[5]Institut Jean Le Rond d'Alembert, Sorbonne Université, CNRS, UMR 7190, F-75 005 Paris, France
[6]Université Paris Cité, CNRS, MSC, UMR 7057, F-75013 Paris, France
[*]Pierre.Suret@univ-lille.fr

ABSTRACT

We report here numerical simulations of Euler equations with the parameters of the experiments described in the Article.

Numerical simulations of Euler equations using High-Order Spectral method

The one-dimensional nonlinear Schrödinger Equation (1DNLSE) used in the Article is established under the assumptions of weak nonlinearity of the wave field as well as the narrowbandedness of its energy content. As it could be expected, at high values of the nonlinear parameter Γ, the measured NDR deviates from the one computed by performing numerical simulations of the 1DNLSE. In order to have a high-fidelity numerical model able to replicate the experiments, we use the direct numerical simulation of the Euler equations solved using the High-Order Spectral (HOS) method[1,2]. Our numerical results have been obtained with the open-source solver HOS-NWT[3]. This model solves the spatio-temporal evolution of the free surface elevation $\eta(\tau,z)$ in a so-called numerical wave tank. It reproduces all the physical features of the experimental (physical) facility including i) the generation of waves by a wave maker and ii) the absorption of those waves when they reach the opposite wall. More details on the HOS-NWT numerical model as well as different validations performed against wave tank experiments can be found in[4,5].

This digital twin is utilized as a complement to the experiments. In the present study, the main advantage of the numerical solution is that it allows to overcome the limitation associated with the finite extent of the physical wave tank (and the associated measuring range $L_m = 120\ m$). As long as the spatial discretization is kept identical, the numerical wave tank can be of arbitrary size. Increasing the size results in a more accurate resolution Δk_{min} for the estimation of wave number k, essential to the evaluation of the NDR and its properties.

In a wave tank environment, numerical and physical experiments start from temporal initial conditions $\eta(\tau = 0, z)$ at rest. Waves are generated by using a wave maker that imposes the spatial initial condition $\eta(\tau, z = 0)$. The HOS digital twin of the École Centrale de Nantes water tank facility (Fig. 2.a) uses the exact same wave maker motions than the experimental ones. This allows the direct deterministic comparison between the experimental and numerical wave probes measurements for validation purposes.

Numerical parameters are chosen after a careful convergence study that ensures the accuracy of the numerical solution. The time integration, achieved thanks to a step-adaptive Runge-Kutta method, is controlled by a tolerance parameter chosen as 10^{-8} for the present long-time integration. An HOS order of nonlinearity set to 5 ensures an accurate numerical solution for all configurations tested. Regarding the spatial discretization, the total length of the numerical domain is set to $L_x = 560\ m$, discretized with $N_x = 12288$ points/modes free of aliasing errors.

Taking into account the evolution of the nonlinear scale z_{nlin} with Γ, the analysis is performed on a length of 531 m for $\Gamma = 0.12$ plotted in Fig. 1. For high nonlinearity $\Gamma = 0.65$, the analysis is conducted on a shorter length of 378 m (see Fig. 1).

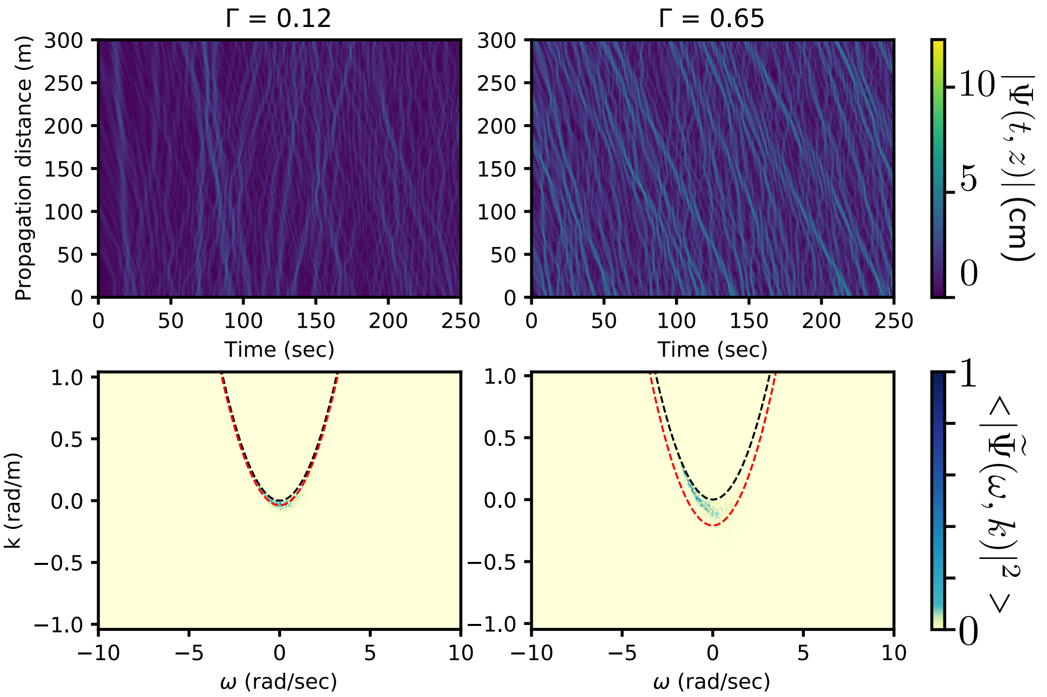

Figure 1. Numerical simulation using high-order spectral method **(top)** Spatiotemporal diagram of the wave envelope amplitude $|\Psi(t,z)|$. **(bottom)** Nonlinear dispersion relation $|\widetilde{\psi}(\omega,k)|^2$ reconstructed from the evolution of the complex wave envelope. **(left)** $\Gamma = 0.12$, $\varepsilon = 0.06$. Simulations are performed for a 531 m long numerical water tank with 177 probes. **(right)** $\Gamma = 0.65$, $\varepsilon = 0.14$. Simulations are performed for a 378 m long numerical water tank with 126 probes.

Similarly to the experimental facility, the numerical results use wave gauges that are equally spaced in the computational domain every 6 *m*.

Overall, the HOS numerical simulations confirm observations in the physical wave tank. As expected, comparing left columns of Fig. 1 and Fig. 1, for small values of Γ, 1DNLSE and HOS results are very similar with a small departure from linear dispersion relation. Then, an increased nonlinearity is associated with coherent structures that are more visible in the (t,z) diagram, see right colmun of Fig. 1. However, compared to the 1DNLSE results (Fig.1 right column), the corresponding straight lines are less frequent in the Euler's simulations. The evolution of those structures is clearly subject to a negative speed when taking into account high-order nonlinear and dispersion effects. The corresponding frequency down-shift is also clearly visible in the NDR.

References

1. Dommermuth, D. G. & Yue, D. K. A high-order spectral method for the study of nonlinear gravity waves. *J. Fluid Mech.* **184**, 267–288 (1987).
2. West, B. J., Brueckner, K. A., Janda, R. S., Milder, D. M. & Milton, R. L. A new numerical method for surface hydrodynamics. *J. Geophys. Res. Ocean.* **92**, 11803–11824 (1987).
3. Ecole Centrale Nantes, LHEEA. Open-source release of HOS-NWT. https://github.com/LHEEA/HOS-NWT.
4. Bonnefoy, F., Ducrozet, G., Le Touzé, D. & Ferrant, P. Time domain simulation of nonlinear water waves using spectral methods. In *Advances in numerical simulation of nonlinear water waves*, 129–164 (World Scientific, 2010).
5. Ducrozet, G., Bonnefoy, F., Le Touzé, D. & Ferrant, P. A modified high-order spectral method for wavemaker modeling in a numerical wave tank. *Eur. J. Mech.* **34**, 19–34 (2012).
